# Superconductivity in high-entropy alloy system containing Th

**DOI:** 10.1038/s41598-023-43085-y

**Published:** 2023-09-28

**Authors:** Piotr Sobota, Rafał Topolnicki, Tomasz Ossowski, Tomasz Pikula, Daniel Gnida, Rafał Idczak, Adam Pikul

**Affiliations:** 1 Institute of Experimental Physics, University of Wrocław, pl. M. Borna 9, 50-204 Wrocław, Poland; 2 Institute of Low Temperature and Structure Research, Polish Academy of Sciences, ul. Okólna 2, 50-422 Wrocław, Poland; 3 Dioscuri Center in Topological Data Analysis, Institute of Mathematics, Polish Academy of Sciences, ul. Śniadeckich 8, 00-656 Warsaw, Poland; 4 Institute of Electronics and Information Technology, Lublin University of Technology, ul. Nadbystrzycka 38A, 20-618 Lublin, Poland

**Keywords:** Superconducting properties and materials, Electronic structure, Structure of solids and liquids, Magnetic properties and materials

## Abstract

Th-containing superconducting high entropy system with the nominal composition (NbTa)$_{0.67}$(MoWTh)$_{0.33}$ was synthesized. Its structural and physical properties were investigated by X-ray diffraction, scanning electron microscopy, energy dispersive X-ray spectroscopy, specific heat, resistivity and magnetic measurements. Two main phases of alloy were observed: major bcc structure and minor fcc. The experimental results were supported by numerical simulation by the DFT Korringa-Kohn-Rostoker method with the coherent potential approximation (KKR-CPA).

## Introduction

High entropy alloys (HEA) can be defined as solid solutions of five or more mixed elements in non-negligible amounts (higher than 5 at.% each), which are characterized by well-defined, ordered crystal structure with high chemical disorder, *i.e.* random distribution of elements at equivalent crystallographic positions. Instead of forming binary, ternary or other intermetallic compounds, they retain simple, closed packed structures similar to those of simple metals. The name ’high entropy’ comes from the large change in configurational entropy during the synthesis of a multi-element alloy, as described by Yeh et al.^1^.

Among other things, the HEAs have become well known as alloys with unusually high mechanical strength and corrosion resistance^2–4^. However, superconducting alloys with high entropy have attracted much attention in recent years. Most of them consists of tantalum-niobium matrices doped with other transition metals, such as Ti, Zr and Hf^5–7^. It has been shown, that chemical composition and molar ratio of the constituent elements strongly affect the properties of the superconducting state (SC) in terms of critical temperature $T_\text{c}$, critical field $H_\text{c}$, and SC transition width^8–12^.

So far, very little is known about actinides-containing high entropy alloys. Currently, HEA with uranium are mainly being studied in the development of advanced high strength materials^13,14^. Recently, however, a superconducting state has also been found in one of the alloys, namely [TaNb]$_{0.31}$(TiUHf)$_{0.69}$^8^, but to the best of our knowledge there are no known superconducting HEAs containing thorium. The main goal of our research is to investigate new high entropy systems with higher critical parameters. Thorium metal would be an interesting addition to Nb-rich HEA, as it has been reported that eutectic Nb-Th system can show much enhanced superconductivity compared to isolated metals^15^. It would also be interesting, from a fundamental research point of view, to study the magnetic behavior of the known HEA superconducting systems when Th is introduced. Thorium possesses 5*f*-electron states, which can cause resulting alloy to exhibit complex magnetic behavior^16–18^. Motivated by this state of affairs, we have undertook a search for the first Th-based superconducting HEA. We chose the recently described superconducting alloy (NbTa)$_{0.67}$(MoHfW)$_{0.33}$^19^ as our starting point. In addition to the synthesis of a new alloy, followed by full structural and physical characterization, we also undertook numerical studies of the electron structure of the obtained alloy. The DFT calculation were also used to explain the formation of more than one phase in studied sample.

## Results and disscusion

### Crystal structure and chemical composition

**Figure 1:**
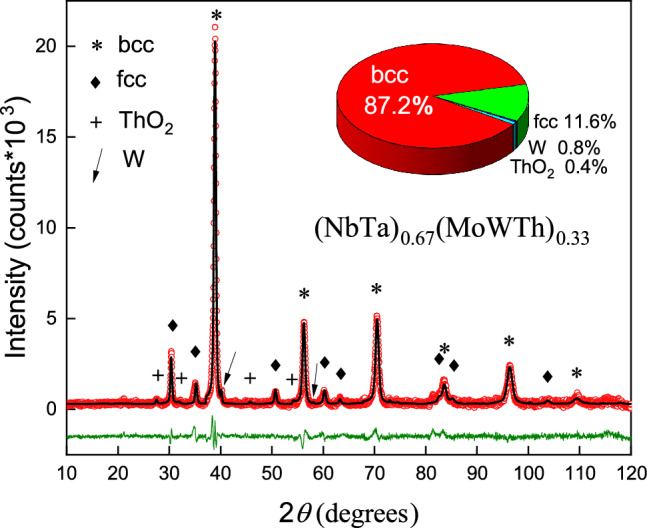
X-ray powder diffraction pattern of (NbTa)$_{0.67}$(MoWTh)$_{0.33}$ along with results of Rietveld refinement of its crystal structure. Red circles and black line represent the experimental data and fit curve, respectively, and green line is the difference between the two. Symbols $*$, $\blacklozenge$, $+$ and $\swarrow$ mark Bragg reflections coming from phases found in the sample and a pie chart shows atomic content of those phases.

**Figure 2:**
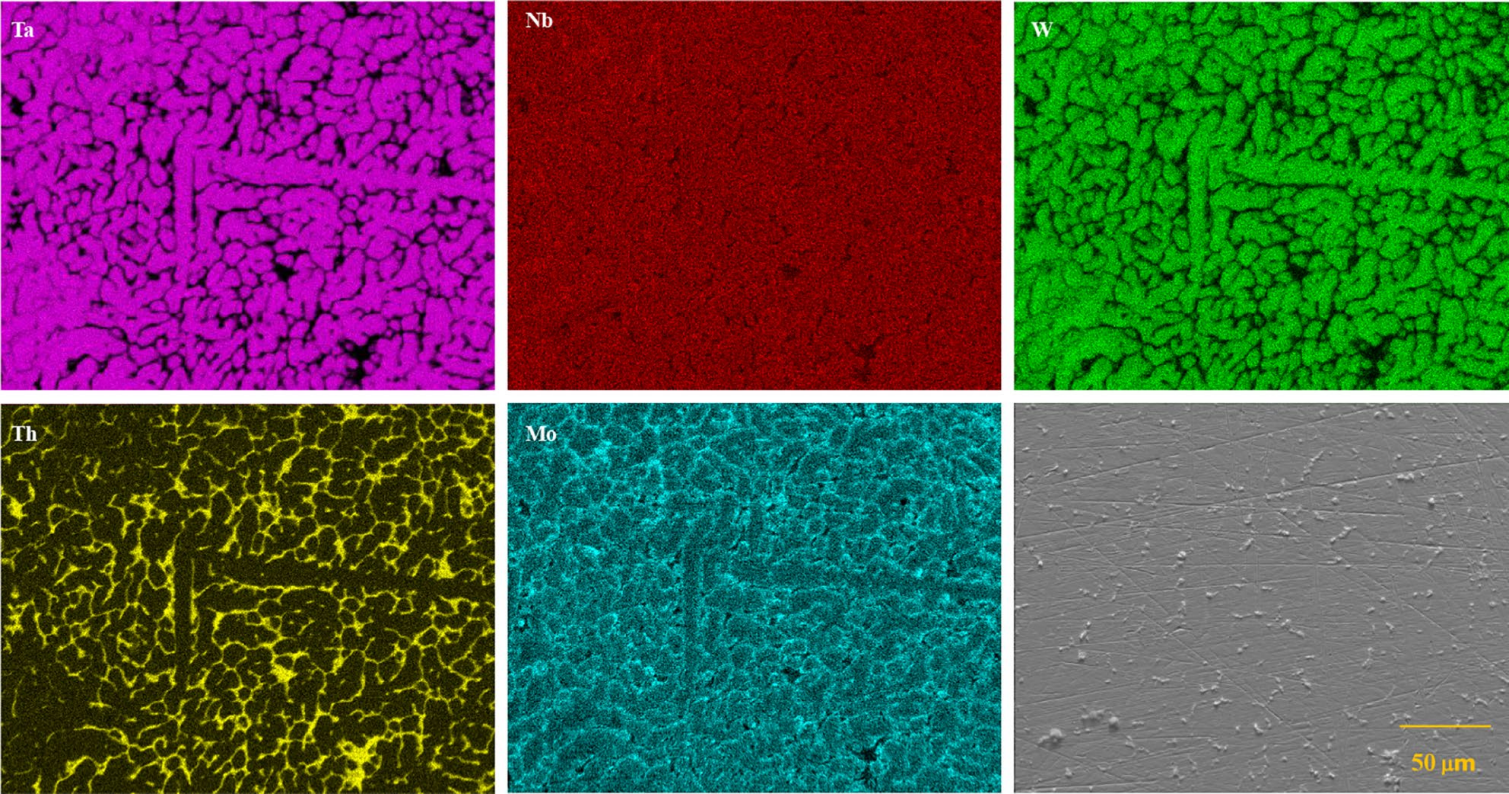
SEM micrographs with EDXS elemental mapping of sample surface of (NbTa)$_{0.67}$(MoWTh)$_{0.33}$. Individual panels are labeled with symbols the content of which was analyzed; panel without the indicated element name shows the sample surface.

Fig. 1 presents powder X-ray diffraction (XRD) patterns obtained for the synthesized alloy (NbTa)$_{0.67}$(MoWTh)$_{0.33}$. Analysis of the experimental data by the Rietveld method showed that the sample consist of four phases: (1) the expected alloy crystallizing in a cubic, body-centered (bcc) structure (87.2 at.%, assuming the atomic distribution from EDS measurements - discussed later in this paper), (2) the expected alloy, but with a face-centred (fcc) structure (11.6 at.%), (3) thorium oxide (0.4 at.%), and (4) tungsten (0.8 at.%). The thorium oxide (ThO$_2$ or thoria) is a refractory oxide with 3573 K melting point, which is a common impurity in thorium-containing compounds and alloys. Its presence in the studied sample was therefore difficult to avoid and, so to speak, expected. The case is similar for tungsten, which has the highest melting points among metals. Therefore, the presence of a small amount of unreacted tungsten is not surprising even with repeated melting of the sample. Fortunately, neither thorium oxide nor free tungsten superconduct and order magnetically (at least in the temperature range studied)^20^, so such small amounts of these impurities had no significant effect on determining the physical properties of the majority phase. However, the influence of the presence of the fcc phase will be discussed in the description of individual properties.

The lattice parameter estimated for the bcc phase was found to be *a* = 3.2725(3) Å. It differs from the lattice parameters of any of the pure metals used in the synthesis, but is close to those of Nb (*a* = 3.3033 Å  according to database Powder Diffraction Data - PDF - ref. no. 34-370) and Ta (*a* = 3.3058 Å  according to PDF 4-788), the two majority elements of the alloy studied. In the case of the fcc phase, *a* is 5.0955(6) Å  which is close to the lattice parameter of pure thorium (*a* = 5.0722 Å  according to PDF 1-920), which also crystallizes in the fcc structure.

Analysis of the chemical composition of the surface of the synthesized sample (Fig. 2) clearly showed presence of two phases with slightly different composition: large grains of the primary (major) phase separated by thin layers of the secondary phase. A closer look at the distribution of individual elements in the sample reveals that the primary phase contains less Th and more Ta and W than the secondary one, while the content of Nb and Mo in both phases is much more similar.

The EDXS analysis of selected spots is shown in Table 1.

**Table 1:** Concentrations of elements in each of the high entropy phases determined by EDS. Four point EDS measurements were made for the primary phase while three for the secondary phase. Average collection area for the primary phase was 83 $\mu m^2$ and 67 $\mu m^2$ for the secondary phase.

Element (at.% )	Nb	Ta	Mo	W	Th
Primary phase	37.8 (±0.9)	36.5 (±1.4)	10.6 (±1.6)	10.4 (±0.7)	4.2 (±1.7)
Secondary phase	30.6 (±2.0)	24.6 (±3.0)	12.1 (±3.1)	7.1 (±1.0)	24.9 (±2.4)

Although EDSX is a semi-quantitative method that provides only approximate data on chemical composition of particular phases (especially when they are not well separated), the data we obtained show that the secondary phase is indeed an alloy, and not just inclusions of pure thorium. In other words, our sample probably contains two HEA alloys, which is consistent with the results of crystal structure refinement mentioned above. Numerical simulation of several structures with various atomic compositions using Density Functional Theory (DFT) methods sheds more light on the possible stoichiometry of the detected alloys (it is discussed later in this paper).

### Physical properties

**Figure 3:**
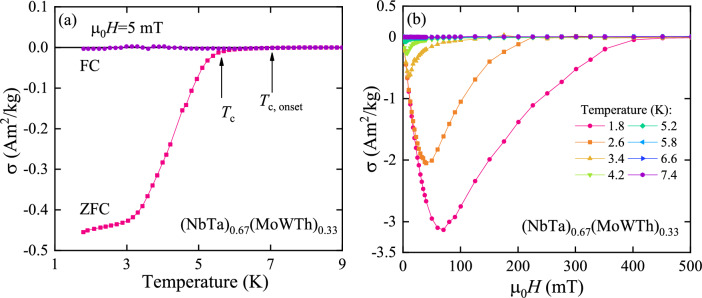
(**a**) Temperature dependence of mass magnetization $\sigma$ of (NbTa)$_{0.67}$(MoWTh)$_{0.33}$ in an external magnetic field $\mu _0 H$, measured at low temperature in both zero-field cooling (ZFC) and field-cooling (FC) regimes; solid curves serve as guides for the eye, and $T_\text{c}$ and $T_\text{c, onset}$ mark superconducting transition in the alloy (see the text for details). (**b**) Field variation of $\sigma$ measured at various temperatures in the superconducting state.

**Figure 4:**
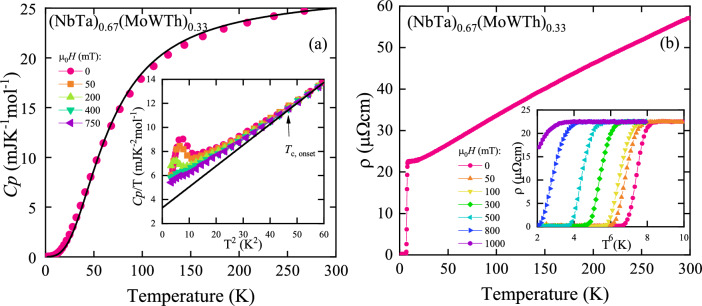
(**a**) Temperature variation of specific heat $C_P$ of (NbTa)$_{0.67}$(MoWTh)$_{0.33}$; insert shows $C_P/$T vs. $T^2$ measured in various external magnetic fields $\mu _0 H$. Black solid curves are fits of Eqs. (1) (main panel) and (2) (insert) to the experimental data. (**b**) Electrical resistivity $\rho$ of the alloy measured as a function of temperature in zero magnetic field; insert shows $\rho (T)$ in various fields. Solid curves serve as guides for the eye.

Measurements of magnetic properties of (NbTa)$_{0.67}$(MoWTh)$_{0.33}$ revealed that it has a weak, featureless and nearly temperature-independent magnetic susceptibility at least down to about 7 K. This means that both the bcc and fcc phase are Pauli-like itinerant paramagnets.

Magnetic measurements in zero field cooling (ZFC) regime (Fig. 3a) shown that sample undergoes transition from paramagnetic state to diamagnetic one at the low temperatures. This transition begins with the onset at about 7 K which starts to develop into sharper decline of mass magnetization $\sigma$ at 5.64(2) K ($T_c$). The sharp decline ends at 3.2 K but diamagnetic signal is progressively getting stronger with cooling. The measurement conducted in field cooling (FC) regime showed no diamagnetic transition, probably due to strong vortex pinning in type-II HEA superconductor. Figure 3b is presenting field dependent magnetisation curves measured at different temperatures. One can see that when temperature rises and disrupt fully developed Meissner state (showed in Fig. 3a as being below 3.2 K), value of $\sigma$ drastically decreases but diamagnetic signal disappears only after heating above 7 K (T$_{c, onset}$).

The $C_p(T)$ dependence of (NbTa)$_{0.67}$(MoWTh)$_{0.33}$ is presented in Fig. 4a. It bears no significant feature above 7 K and it follows Dulong- Petit law at RT. Experimental points of the curve measured in nominal field $\mu _0 H$=0 K were easily described by the conventional formula:

1 $$\begin{aligned} C_P(T) = \gamma T + 9 R r\left( \frac{T}{\Theta _\text{D}}\right) ^{3}\int _0^{\Theta _\text{D}/T}\frac{x^4 e^x}{\left(e^x - 1\right)^2}dx, \end{aligned}$$

in which the first term describes the electron contribution to the specific heat according to the Sommerfeld model, and the second term is the phonon contribution to $C_P(T)$ in terms of the Debye model, with the Sommerfeld coefficient $\gamma$ and Debye temperature $\Theta _\text{D}$, as fitting parameters; *R* denotes the universal gas constant and *r* is the number of atoms in the formula unit, which in this case is equal to 1. Results show simple metallic behaviour of this alloy at the normal state. Insert in Fig. 4a presents $C_p/$T measured at several selected fields at low temperatures in function of $T^2$, reviling two inclines corresponding to T$_{c, onset}$ and $T_{c}$ previously observed in Fig. 3a. The level of incline is relatively small in comparison to $\lambda$-shaped superconductive transition below $T_{c}$.

Below 7 K function $C_P/T$ of (NbTa)$_{0.67}$(MoWTh)$_{0.33}$ in the normal state can be described by the $T^3$-Debye law (bold black line in Fig. 4a).:

2 $$\begin{aligned} \frac{C_P (T)}{T} = \gamma + \beta T^2, \end{aligned}$$

with $\gamma$ = 3.31(4) mJ K$^{-2}$ mol$^{-1}$ and $\beta$ = 0.1739(5) mJ K$^{-4}$ mol$^{-1}$. Due to the presence of a non-negligible amount of fcc phase both of those parameters should be treated as an average of two phases strongly dominated by the bcc phase. Using lattice specific heat coefficient $\beta$ as fitting parameter to equation:

3 $$\begin{aligned} {\Theta _\text{D}^\text{LT}=\left( n\frac{12R \pi ^4}{5\beta } \right) ^3}, \end{aligned}$$

specific Debye temperature ${\Theta _\text{D}^\text{LT}}$=223.59(2) K was found. The $T_c$ and ${\Theta _\text{D}^\text{LT}}$ were used to calculate electron-phonon coupling from McMillan’s equation^21^:

4 $$\begin{aligned} \lambda _\mathrm{el-ph}=\frac{1.04+\mu ^{*}\ln \left( \frac{\Theta _\text{D}^\text{LT}}{1.45 T_\text{c}} \right) }{\left( 1-0.62 \mu ^{*} \right) \ln \left( \frac{\Theta _\text{D}^\text{LT}}{1.45 T_\text{c}} \right) -1.04 }, \end{aligned}$$

where $\mu ^{*}$ is the Coulomb repulsion constant. Taking $\mu ^{*}$ = 0.125 (a value commonly used for systems containing mostly *d*-electron elements), we obtained $\lambda _\mathrm{el-ph}$ = 0.72(4). That classifies this alloy in the range of intermediate electron-phonon coupled superconductors. To estimate conductive electron density of states at the Fermi level $N(E_\text{F})$ = 1.4, Sommerfeld coefficient $\gamma$ was used in following equation:

5 $$\begin{aligned} \gamma = \frac{1}{3} \pi ^{2} k_\text{B}^2 N_\text{A} N(E_\text{F}) \end{aligned}$$

Then, density of non-interacting electrons $N(E_\text{F})^*$ = 0.8(1)  states eV$^{-1}$ f.u.$^{-1}$ was calculated based on following equation^22^:

6 $$\begin{aligned} N(E_\text{F})^* = \frac{N(E_\text{F})}{1+ \lambda _\mathrm{el-ph}}. \end{aligned}$$

Fig. 4b depicts electrical resistivity  $\rho$ measured in nominal zero applied field. As shown, the electrical resistivity decreases with decreasing temperature in a manner characteristic of metals. At low temperatures the resistivity drops to zero, indicating superconducting properties of the compound under investigation. Here we define $T_c$ at 10% of residual resistivity, which in nominal field is 7 K. The RRR ratio is equal to 2.54, and its low value is a result of the polycrystalline nature and high degree of structural disorder in the sample. Insert in Fig. 4b shows temperature dependent curves measured in various fields. Transition to zero resistance is visible even under applied nominal field higher than used in other methods.

**Figure 5:**
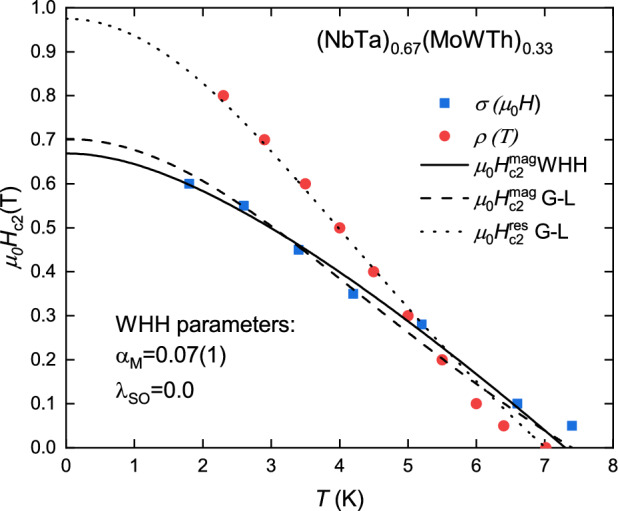
Phase diagram of superconducting (NbTa)$_{0.67}$(MoWTh)$_{0.33}$ obtained from magnetization and resistivity measurements. Dotted and dashed lines are obtained from Eq. (7) while solid line is an experimental fit to the WHH model.

The experimental curves of $\mu _0 H_\text{c2}(T)$ obtained for (NbTa)$_{0.67}$(MoWTh)$_{0.33}$ can be described by the Ginzburg-Landau (G-L) equation:

7 $$\begin{aligned} \mu _0 H_{\text{c}2}(T) = \mu _0 H_{\text{c}2} (0) \frac{1-\left( T/T_\text{c}\right) ^2}{1+\left( T/T_\text{c}\right) ^2} \end{aligned}$$

yielding $\mu _0 H_\text{c2}^\text{mag}(0) = 0.73(4)$ T (for data from Fig. 3b) and $\mu _0 H_\text{c2}^\text{res}(0) = 0.98(2)$ T (for data from Fig. 4b) as the least-squares fitting parameters (see the dashed and dotted lines in Fig. 5). The $\mu _0 H_\text{c2}^\text{mag}(0)$ was also estimated using the full Werthamer-Helfand-Hohenberg (WHH) formalism for isotropic-gap BCS superconductors in the dirty limit, incorporating the spin-paramagnetic effect via the Maki parameter $\alpha _\text{M}$ and the spin-orbit scattering constant $\lambda _\text{SO}$^23–25^:

8 $$\begin{aligned} \ln \frac{1}{t}=\bigg (\frac{1}{2} +\frac{i\lambda _{\text{SO}}}{4\gamma } \bigg ) \psi \bigg ( \frac{1}{2} + \frac{\overline{h}+\frac{1}{2}\lambda _{\text{SO}} +i\gamma }{2t}\bigg ) + \bigg (\frac{1}{2} -\frac{i\lambda _{\text{SO}}}{4\gamma } \bigg ) \psi \bigg ( \frac{1}{2} + \frac{\overline{h}+\frac{1}{2}\lambda _{\text{SO}} -i\gamma }{2t}\bigg ) - \psi \bigg (\frac{1}{2}\bigg ) \end{aligned}$$

where $\gamma \equiv \sqrt{(\alpha _\text{M} \overline{h})^2 - (\frac{1}{2} \lambda _{\text{SO}})^2}$, $\overline{h} = \frac{4}{\pi ^2} \frac{H_{\text{c2}}}{-dH_{\text{c2}}/dT}$ and $t=\frac{T}{T_C}$. Best fit to the experimental data was found for $\lambda _\text{SO}$ equal to zero, what suggest that spin-orbit scattering has very weak effect on the value of the upper critical field. The value of Maki parameter in aforementioned fit was 0.07(1), and the $\mu _0 H_\text{c2}^\text{mag}(0)$ obtained from WHH model was equal to 0.67(1) T In order to determine the pair-braking mechanism, the orbital upper critical field $\mu _0 H_\text{c2}^\text{orb}$=0.67(1) T (virtually equal to $\mu _0 H_\text{c2}^\text{mag}(0)$ from WHH model) was estimated using $\mu _0 H_\text{c2}^\text{mag}(0)$ data and the formula developed for type-II superconductors in the dirty limit scenario^24,25^:

9 $$\begin{aligned} \mu _0 H_\text{c2}^\text{orb}=-0.693 \times T_\text{c} \left[ \text{d}(\mu _0 H_\text{c2}(T))/\text{d}T \right] _{T = T_\text{c}} \end{aligned}$$

where the value of $\left[ \text{d}(\mu _0 H_\text{c2}(T))/\text{d}T \right] _{T = T_\text{c}}$ is given by the formula:

10 $$\begin{aligned} \left[ \text{d}(\mu _0 H_\text{c2}(T))/\text{d}T \right] _{T = T_\text{c}} = \frac{\alpha _\text{M}}{-0.528}. \end{aligned}$$

The Pauli limiting field $\mu _0 H_\text{P}$= 13.4(2) T was estimated from the equation^26^:

11 $$\begin{aligned} \mu _0 H_\text{P} = 1.84T_c. \end{aligned}$$

According to Eq. (2) electronic contribution $\textit{C}_{el}$ to the specific heat can be estimated by simply subtracting $\beta$ $\textit{T}^3$ - the lattice contribution. The resulting $\textit{C}_{el}$(T) dependency can be described using the formula from the Bardeen-Cooper-Schrieffer (BCS) theory of superconductivity:

12 $$\begin{aligned} C_\text{BCS}(T) = A \gamma T_\text{c} \exp \left( -\frac{\Delta _{0}}{k_\text{B}T} \right) , \end{aligned}$$

where $\Delta _{0}$ is a superconducting energy gap, and *A* is a constant. The least-squares fit of this equation to the experimental data yielded $\Delta _{0}/k_\text{B}$ = 7.4(3) K. Using the value of superconductive gap and Sommerfeld coefficient per volume (derived from the known mass of the sample and the estimated density of the alloy equal to 12903 kgm$^{-3}$) $\gamma _{v}$=304.6(3.7) J m$^{-3}$K$^{-2}$, it is possible^27^ to calculate the thermodynamic critical field $\mu _{0}H_\text{c}(0)$:

13 $$\begin{aligned} \mu _{0}H_\text{c}(0)=\sqrt{\frac{3 \gamma _{V}}{2\pi ^{2}\mu _{0}}} \frac{\Delta _{0}}{k_\text{B}}, \end{aligned}$$

as equal to 28.4(1) mT.

Using the values derived from Eqs. (9) and (13) it is possible to estimate the Ginzburg-Landau coherence length $\xi _\text{GL}$^28^:

14 $$\begin{aligned} \xi _\text{GL} =\sqrt{\frac{\phi _{0}}{2\pi \mu _{0}H_\text{c2}^\text{orb}(0)}} \end{aligned}$$

and the Ginzburg-Landau penetration depth $\lambda _\text{GL}$:

15 $$\begin{aligned} \lambda _\text{GL} = \sqrt{\frac{\phi _0 \mu _0 H_\text{c2}^\text{orb}(0)}{4\pi \mu _0 H_\text{c}(0)^2}}. \end{aligned}$$

For the studied alloy, these values are $\xi _\text{GL}$ = 25.13 nm and $\lambda _\text{GL}$ = 326.52 nm, respectively. From them the Ginzburg-Landau parameter $\kappa _\text{GL}$, defined as:

16 $$\begin{aligned} \kappa _\text{GL} = \frac{\lambda _\text{GL}}{\xi _\text{GL}}, \end{aligned}$$

12.99 is derived.

The lower critical field $\mu _0 H_\text{c1}(0)$ was estimated to be 3.4 mT and 4.0 mT respectively, using the equations^29^:

17 $$\begin{aligned} \mu _0 H_\text{c1}=\mu _0 H_\text{c2}^\text{orb}\frac{ln(\kappa _\text{GL})+\alpha (\kappa _\text{GL})}{2\kappa _\text{GL}^2} \end{aligned}$$

and

18 $$\begin{aligned} \mu _0 H_\text{c1}=\frac{\phi _0}{4\pi \lambda _\text{GL}^2}\left[ln(\kappa _\text{GL})+\alpha (\kappa _\text{GL})\right] \end{aligned}$$

where $\alpha$ is calculated from^29^.

From the collected data regarding superconducting state it is evident that this alloy posses two superconductive phases - one with $T_c$ around 7 K and other with $T_c$ at 5.6 K. The latter is most likely corresponding to the bulk, Th-depleted, bcc phase as its transition temperature is close to the other HEA of similar composition. Also depth of Meissner state as well as phase transition peak in $C_p/$T curve indicates that this is the signal of dominant phase. Overlapping of the phases signals makes it impossible to determine the exact $T_c$ of bcc phase from $C_p$/T curve. On the other hand the diamagnetic signal of the phase with higher $T_c$ depicted in Fig. 3 has only minimal incline so $T_c$ of the main phase could be described as above. In the $C_p$/T high $T_c$ phase transition is extremely broad. It is hard to attribute this to Th-rich phase crystallizing in fcc structure as one would expect sharper transition due to sheer amount of this phase. Instead, based on SEM-EDS observations, these signals could originate from minuscule phases with disturbed stochiometry that could be formed on the interphase between well crystallized, superconductive bcc phase and nonsuperconductive fcc phase as both of them are not well separated, but merging one into another. That would explain weak but broad transitions seen in Figs. 3 and 4a but also robust electrical resistance in Fig. 4b. Unusual width of transition in $C_p$/T can sometimes be related to a specific chemical composition^9,19^ but the inhomogeneity of the phases (seen in SEM-EDS micrographs) is most likely the case in this system. A link between mesoscopic inhomogeneity and such broad transitions have recently been reported in Ti-Hf-Nb-Ta-Re^30^ and Sc-Hf-Nb-Ta-Ti-Zr^31^ systems. However, the formation of nanostrucutres does not seem to affect the superconductive properties of HEA^32^. To further analyze the two phases in regards of their stability and superconductive properties, the DFT numerical simulations were performed.

### Numerical simulations

**Figure 6:**
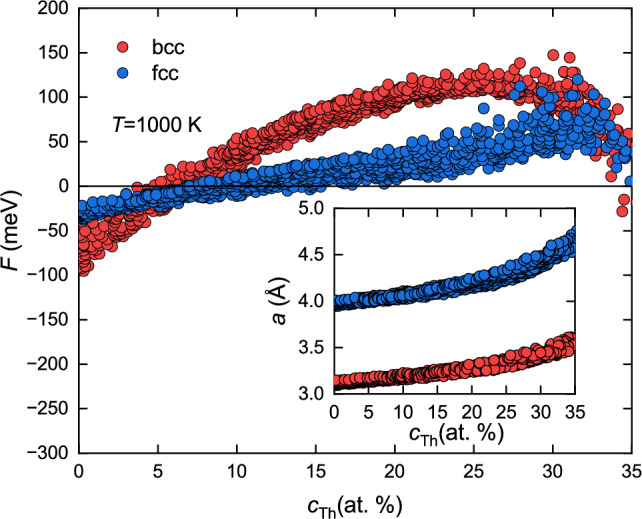
Free energy changes calculated for both bcc and fcc phases in as a function of Th concentration; insert shows the equilibrium lattice parameter determined from the relaxation of the unit cell.

The experimental data evidently proved that our (NbTa)$_{0.67}$(MoWTh)$_{0.33}$ sample contains two HEA. The major Th-depleted phase crystallizes in bcc structure while the secondary Th-rich phase crystallizes in fcc. In order to study the relative stability of bcc vs. fcc phases, the total energy computations were done using KKR-CPA method for these two phases varying the concentrations of the constituent elements of the alloy around their experimentally observed stechiometry. For each structure and composition, the equilibrium lattice parameter were derived from the relaxation of the unit cell. The free energy *F* at temperature *T* for bcc and fcc phases was computed using equation^33^:

19 $$\begin{aligned} F = E_\text{form} - TS = E_\text{alloy} - \sum _{i=1}^{n}{c_i E_i} - TS, \end{aligned}$$

where the ground state formation energy ($E_\text{form}$) is the difference between the total energy of a given alloy $E_\text{alloy}$ and sum of total energies of elemental bulks $E_i$, weighted over concentrations $c_i$, while *S* is the total entropy. In the case of HEA, *S* is dominated by the configurational entropy $S_\text{conf}$ which in random solid system containing *n* elements is given by combinatorial formula^1^:

20 $$\begin{aligned} S \approx S_\text{conf} = -k_\text{B} \sum _{i=1}^{n}{c_i \ln c_i}. \end{aligned}$$

The calculations of *F* at $T=0,300, 1000$ and 2000 K were performed for over 1000 bcc and 1000 fcc HEA structures in which the atomic concentration of each element in the NbTaMoWTh alloy varies in the range of 10% - 46% for Ta, 10% - 49% for Nb, 0.2% - 22% for Mo, 0% - 17% for W and 0% - 35% for Th. Compositions of considered phases were constructed randomly in a process similar to the method of acceptance-rejection sampling, where the atomic concentrations are drawn randomly for each element in the ranges mentioned above. If the concentrations summed up to 100% (with 1% margin) CPA-KKR calculations were performed. Insert in the Fig. 6 shows the equilibrium lattice parameters and *F* values computed for all taken into account HEA structures as function of Th concentration ($c_\text{Th}$). The results presented here are for $T=1000$ K, for other temperatures see the online supplementary information Figs. S1–S3. Since the crystal stability of the system is related to the minimum of the free energy, the obtained results show the preference of bcc structures for $c_\text{Th}<5\%$, while fcc one for $c_\text{Th}$ between 5% and 32%. These findings are in agreement with XRD and SEM results which reveal the coexistence of bcc HEA phase with Th content close to 4 at.% and fcc HEA phase with about 25 at.% of Th. For higher Th concentrations ($32\%<c_\text{Th} \le 35\%$), the difference of *F* derived for both considered structures becomes relatively small indicating that the bcc and fcc phases may coexist together (heterogeneous equilibrium). Alternative approach to the computation of the formation energy is described in the supplementary information. Results are presented on Supplementary Figs. S4–S7.

**Figure 7:**
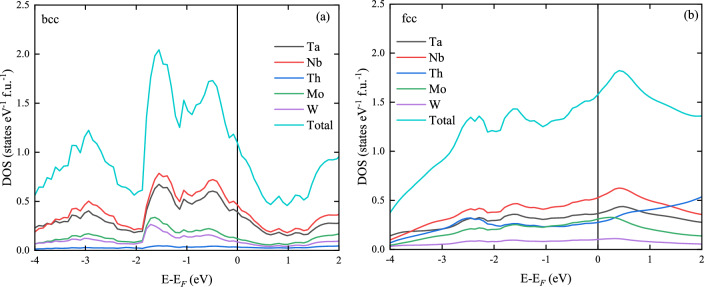
Electron density of states of of (**a**) bcc Nb$_{0.38}$Ta$_{0.37}$Mo$_{0.11}$W$_{0.10}$Th$_{0.04}$ and (**b**) fcc Nb$_{0.31}$Ta$_{0.25}$Mo$_{0.12}$W$_{0.07}$Th$_{0.25}$ alloys calculated by the KKR-CPA method. Total and partial atomic densities, color coded and weighted by their atomic concentrations.

The electronic band structures for all considered bcc and fcc NbTaMoWTh alloys were calculated using the KKR-CPA method. In the first step, we choose two particular HEA compositions (bcc Nb$_{0.38}$Ta$_{0.37}$Mo$_{0.11}$W$_{0.10}$Th$_{0.04}$ and fcc Nb$_{0.31}$Ta$_{0.25}$Mo$_{0.12}$W$_{0.07}$Th$_{0.25}$) which are close to those estimated using EDXS analysis. Total and partial atomic densities of states (DOS) of these two alloys are shown in Fig. 7. In the case of bcc alloy the shape of the total density of states (TDOS) is similar to this reported for the superconducting bcc HEA (NbTa)$_{0.67}$(MoHfW)$_{0.33}$^19^. The Fermi level lies 0.54 eV above the nearest TDOS maximum while the largest contribution to TDOS in the Fermi-level region ($E - E_\text{F}$ from -4 to 2 eV) comes from Nb and Ta atoms, due to their highest atomic concentrations in the alloy. For fcc Nb$_{0.31}$Ta$_{0.25}$Mo$_{0.12}$W$_{0.07}$Th$_{0.25}$, one can note larger TDOS at the Fermi energy $N^\text{KKR}(E_\text{F})$ = 1.57  states eV$^{-1}$ f.u.$^{-1}$ than 1.09  states eV$^{-1}$ f.u.$^{-1}$ for the bcc Nb$_{0.38}$Ta$_{0.37}$Mo$_{0.11}$W$_{0.10}$Th$_{0.04}$. Since the DFT calculations which are performed in this work take into account only the non-interacting electrons, the obtained $N^\text{KKR}(E_\text{F})$ values can be compared with experimentally determined $N(E_\text{F})^*$ = 0.8(1)  states eV$^{-1}$ f.u.$^{-1}$. As one can notice, the theoretical value of $N^\text{KKR}(E_\text{F})$ for bcc HEA is slightly higher than experimental one. In the case of fcc HEA, $N^\text{KKR}(E_\text{F})$ is much higher than $N(E_\text{F})^*$ and is similar to $N(E_\text{F})$. Therefore, taking into account Eq. (6) this result suggests that for fcc phase, $\lambda _\mathrm{el-ph}$ could be close to 0.

In the next step, taking the calculated $N^\text{KKR}(E_\text{F})$ values for all considered bcc and fcc NbTaMoWTh alloys, it is possible to predict the theoretical value of superconducting critical temperature ($T_\text{c}^\text{KKR})$. For each alloy $T_\text{c}^\text{KKR}$ was estimated using McMillan’s formula (Eq. (4)). The Coulomb repulsion constant $\mu ^{*}$ was obtained from the empirical relation^34^:

21 $$\begin{aligned} \mu ^{*} = \frac{0.26 N^\text{KKR}(E_\text{F})}{1+N^\text{KKR}(E_\text{F})}. \end{aligned}$$

**Figure 8:**
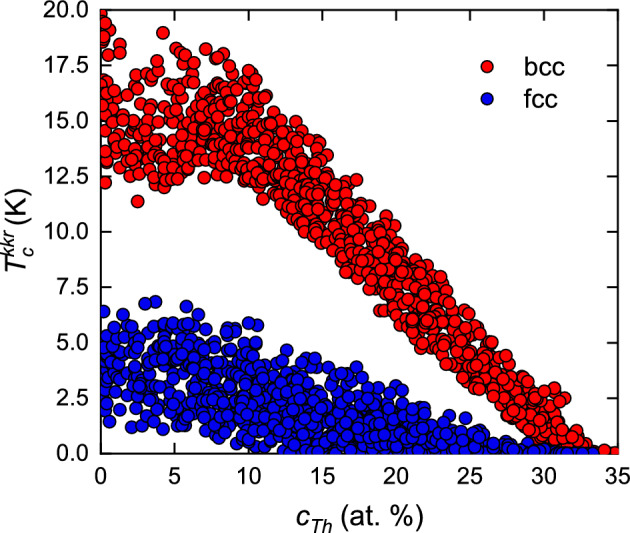
Theoretical predictions of superconducting critical temperature ($T_\text{c}^\text{KKR}$) for various NbTaMoWTh alloys containing up to 35 at.% of Th.

${\Theta _\text{D}^\text{LT}}$ was calculated from the relation given by Moruzzi et al.^35^:

22 $$\begin{aligned} \Theta _\text{D}^\text{LT} = 41.63 \sqrt{\frac{S_0 B}{M}}, \end{aligned}$$

where *B* is the bulk modulus evaluated at the equilibrium Wigner-Seitz sphere radius $S_0$ and *M* is the atomic mass of given HEA. Finally, $\lambda _\mathrm{el-ph}$ parameter was estimated as follows^19,22,36^:

23 $$\begin{aligned} \lambda _\mathrm{el-ph} = \frac{\gamma _\text{exp}}{\gamma _\text{th}}-1 = \frac{\sum _{i=1}^{n}{c_i \gamma _i}}{\gamma _\text{th}}-1, \end{aligned}$$

where the theoretical specific heat coefficient $\gamma _\text{th}$ was obtained by inserting $N^\text{KKR}(E_\text{F})$ value into relation Eq. (5) and the experimental specific heat coefficient $\gamma _\text{exp}$ was approximated by a sum of specific heat coefficients of elemental bulks $\gamma _i$, weighted over concentrations $c_i$. The computed values of $T_\text{c}^\text{KKR}$ for over 1000 bcc and 1000 fcc NbTaMoWTh structures containing up to 35 at.% of Th are presented in Fig. 8. It should be noted here that in the case of superconducting HEA, the critical temperatures calculated using KKR-CPA method are generally two time higher than the experimental ones. In particular, for Ta$_{0.34}$Nb$_{0.33}$Hf$_{0.08}$Zr$_{0.14}$Ti$_{0.11}$ HEA, the experimental $T_\text{c}$ = 7.3 K while theoretically predicted $T_\text{c}^\text{KKR}$ = 15 K^36^. For (NbTa)$_{0.67}$(MoHfW)$_{0.33}$, $T_\text{c}$ = 4.3 K and $T_\text{c}^\text{KKR}$ = 8.8 K^19^. Since that, the results presented in Fig. 8 should be treated as a crude estimate of real critical temperatures. However, some general trends can be discussed. Firstly, up to $c_\text{Th}=30\%$, $T_\text{c}^\text{KKR}$ calculated for bcc HEA are much higher than for fcc alloys with the same atomic composition. Secondly, above $c_\text{Th}=10\%$, $T_\text{c}^\text{KKR}$ values rapidly decrease with Th concentration. This tendency is much more pronounced in the case of bcc HEA. Connecting these results with experimental data, one can state that the broad superconducting transition observed in magnetic and specific heat measurements is caused be inhomogeneous distribution of Th atoms in the bcc system which leads to a certain distribution of the $T_\text{c}$ values. Finally, dividing $T_\text{c}^\text{KKR}$ values by factor of 2, one can obtain the mean critical temperature for bcc HEA with $c_\text{Th}<10\%$ close to 7-8 K which is comparable with $T_\text{c, onset}$ observed experimentally, while for fcc HEA with $c_\text{Th} \approx 25\%$ the mean critical temperature is close to 0 K and again this result is in agreement with experimental data.

## Conclusions

The synthesis and physical properties of the high-entropy system (NbTa)$_{0.67}$(MoWTh)$_{0.33}$ were described. Refinement of the crystal structure as well as SEM imaging and mapping shows segregation on two major phases, one rich in Th (crystallizing in fcc structure) and other Th-depleted (crystallizing in bcc structure) as well as W and ThO$_2$ impurities. Specific heat, magnetic and resistivity measurements let ascribe superconductive state with $T_c$ = 5.64(2) K to bcc structure, while $T_{c,onset}$ around 7 K as originating from minuscule, distorted phases forming on an interphase of fcc and bcc structures. This interpretation was supported by DFT numerical simulations which reveal that SC state in fcc phase would be very unlikely.

## Methods

### Synthesis

A polycristalline (NbTa)$_{0.67}$(MoWTh)$_{0.33}$ sample was synthesised by conventional arc melting technique where the stochiometric amounts of pure elements and Ti-gettered Ar atmosphere were used. Process was repeated eight times. To ensure minimal amount of ThO$_2$ in thorium metal, the raw material was polished with metal file and remelted few times before the synthesis of the alloy. The total weight loss after the synthesis was less than 0.3$\%$.

### X-ray diffraction

Crystal structure of the product was studied by powder X-ray diffraction (XRD) using a PANalytical X’pert Pro diffractometer with CuK$\alpha$ radiation. The experimental XRD pattern was analyzed by the Rietveld method using the HighScore Plus software.

### Elemental analysis

Chemical composition and phase composition of the sample were verified by energy dispersive X-ray spectroscopy (EDXS) using a FESEM FEI Nova NanoSEM 230 scanning electron microscope equipped with an EDAX Genesis XM4 spectrometer on polished surface of the cut specimen.

### Magnetic measurments, heat capacity and electrical resistivity

Magnetic properties of the alloy were studied in temperature range 1.72–300 K and in magnetic fields up to 20 kOe using a commercial Quantum Design MPMS-XL magnetometer. Heat capacity and electrical resistivity were measured from room temperature down to 1.8 K using a Quantum Design PPMS platform.

### Theoretical calculations

Free energy and electronic structure calculations were performed using the Korringa-Kohn-Rostoker (KKR) method, which in the case of disordered systems was implemented together with the coherent potential approximation (CPA), where a random arrangements of all elements is replaced by the ordered lattice representing an average over all possible configurations of the disordered lattice within the simple unit cell (bcc or fcc)^37–41^. In this study, the KKR-CPA method implemented in the AkaiKKR (MACHIKANEYAMA) package was used^42–44^. The Perdew-Burke-Ernzerhof exchange-correlation functional (PBE) was applied to construct the muffin-tin crystal potential in the semirelativistic approach^45,46^. The cutoff for the angular momentum was set to $l_\text{max}$ = 3 and 256 *k* points were used to sample the irreducible part of the Brillouin zone during the self-consistent cycle and density of states calculations. Additionally, values up to 5216 *k* points were tested yielding virtually the same results. Atomic sphere approximation (ASA) was utilized in all calculations. For each considered HEA structure and composition, the lattice parameter *a*, bulk modulus *B* and its derivative $B'$ of the crystal were derived using the Murnaghan equation of state^47^.

### Supplementary Information

Supplementary Information.

## Data Availability

The data presented in this study are openly available in OSF repository at DOI 10.17605/OSF.IO/G4N6B (subsection: Superconductivity in high-entropy alloy system containing Th).
