# Supplementary material for: Superconductivity in high-entropy alloy system containing Th
Source: Sci Rep. 2023 Sep 28;13:16317. doi: 10.1038/s41598-023-43085-y (PMC10539351; doi:10.1038/s41598-023-43085-y)
Supplement: Supplementary file 1 — Supplementary Information. [file 41598_2023_43085_MOESM1_ESM.pdf]

Supplementary information to: Superconductivity in high-entropy alloy system containing Th

Piotr Sobota*[1,2], Rafał Topolnicki[1,3], Tomasz Ossowski[1], Tomasz Pikula[4], Daniel Gnida[2], Rafał Idczak[1] and Adam Pikul[2]

[1]Institute of Experimental Physics, University of Wrocław, pl. M. Borna 9, 50-204 Wrocław, Poland.

[2]Institute of Low Temperature and Structure Research, Polish Academy of Sciences, ul. Okólna 2, 50-422 Wrocław, Poland.

[3]Dioscuri Center in Topological Data Analysis, Institute of Mathematics, Polish Academy of Sciences, ul. Śniadeckich 8, 00-656 Warsaw, Poland.

[4]Institute of Electronics and Information Technology, Lublin University of Technology, ul. Nadbystrzycka 38A, 20-618 Lublin, Poland.

*piotr.sobota2@uwr.edu.pl.

Free energy changes calculated for both bcc and fcc phases in as a function of Th concentration at T = 0, 300, and 2000 K are presented in Figures S1-S3. The calculations of F were performed for over 1000 bcc and 1000 fcc HEA structures in which the atomic concentration of each element in the $(NbTa)_{0.67}(MoWTh)_{0.33}$ alloy varies in the range of 10% - 46% for Ta, 10% - 49% for Nb, 0.2% - 22% for Mo, 0% - 17% for W and 0% - 35% for Th.

The formation energy of the separated phases can be computed, assuming decomposition of nominal phase into two well defined phases, using the formula:

$$\Delta E_{nom} = p_{bcc}E_{bcc} + p_{fcc}E_{fcc} - E_{nominal} \tag{1}$$

where p_{bcc} is as amount of bcc phase, $p_{fcc} = (1 - p_{bcc})$ is an amount of FCC phase and E_{bcc}/E_{fcc} is their corresponding formation energies.

The ΔE_{nom} measures the energetic gain of splitting the nominal phase -$(NbTa)_{0.67}(MoWTh)_{0.33}$- into two separated phases: if it is negative then splitting the nominal phase into two phases is energetically favorable. Here the BCC and FCC cannot have arbitrary composition as for fixed p_{bcc} one needs to recover the composition of the nominal phase. In other words for given stoichiometry of BCC phase and fixed p_{bcc} there might exist at most one composition of FCC phase. In our additional calculations we set the p_{bcc} to 0.90 and for each BCC phase had in our study we calculated composition of the corresponding FCC phase (which ensures overall distribution of elements as in nominal phase). Next, the optimal lattice constant for this FCC phase is determined and its formation energy is computed using equation:

$$F = E_{\text{form}} - TS = E_{\text{alloy}} - \sum_{i=1}^{n} c_i E_i - TS, \tag{2}$$

Afterwards the ΔE_{nom} is computed for this pair of BCC and FCC structures. The procedure was then repeated for $p_{bcc} = 0.85$ and $p_{bcc} = 0.80$. The experiment was conducted in T=0K (when configuration entropy is neglected), in T=300K, 1000K, and 2000K (in the case of the latter three the formation energy becomes formation-free energy, but for simplicity, we will denote it by E). Results are depicted in supplementary Figures S4-S7. As can be seen, the energetic gain increases (ΔE_{nom} becomes more negative) as Th concentration in the BCC phases decreases. The conclusion is the same for all considered temperatures.

Supplementary information to: Superconductivity in high-entropy alloy system containing T

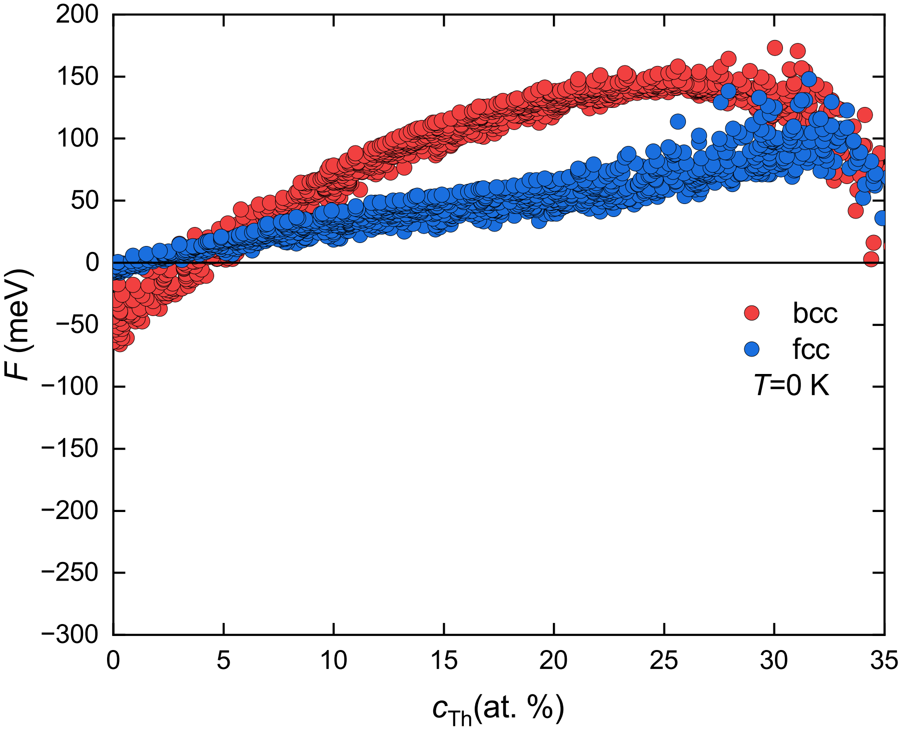

Figure S1 Free energy changes calculated for both bcc and fcc phases in as a function of Th concentration at the temperature of 0 K.

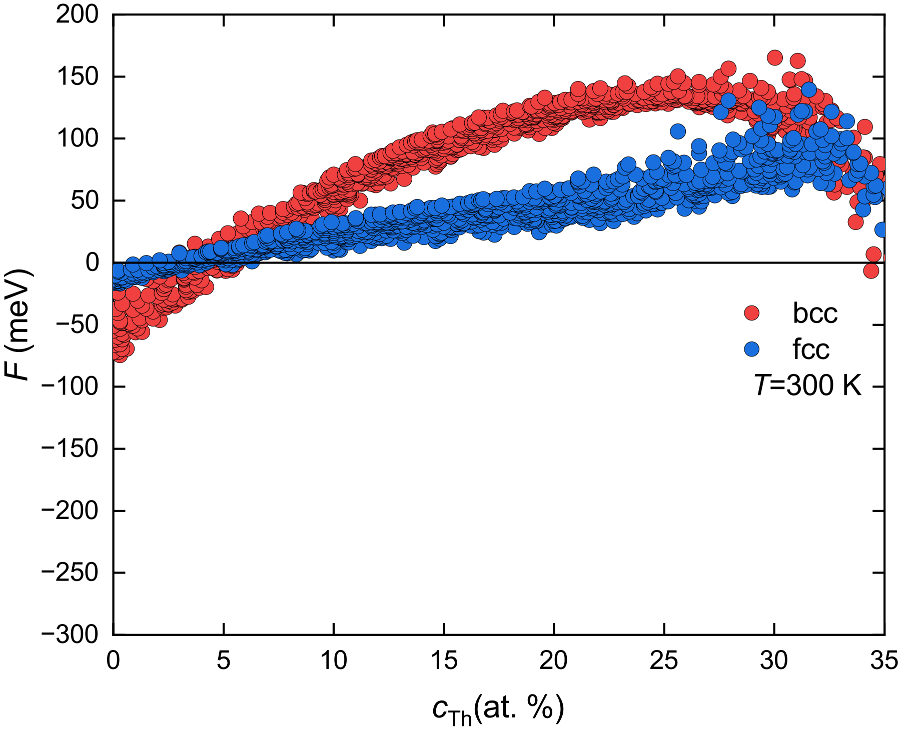

Figure S2 Free energy changes calculated for both bcc and fcc phases in as a function of Th concentration at the temperature of 300 K.

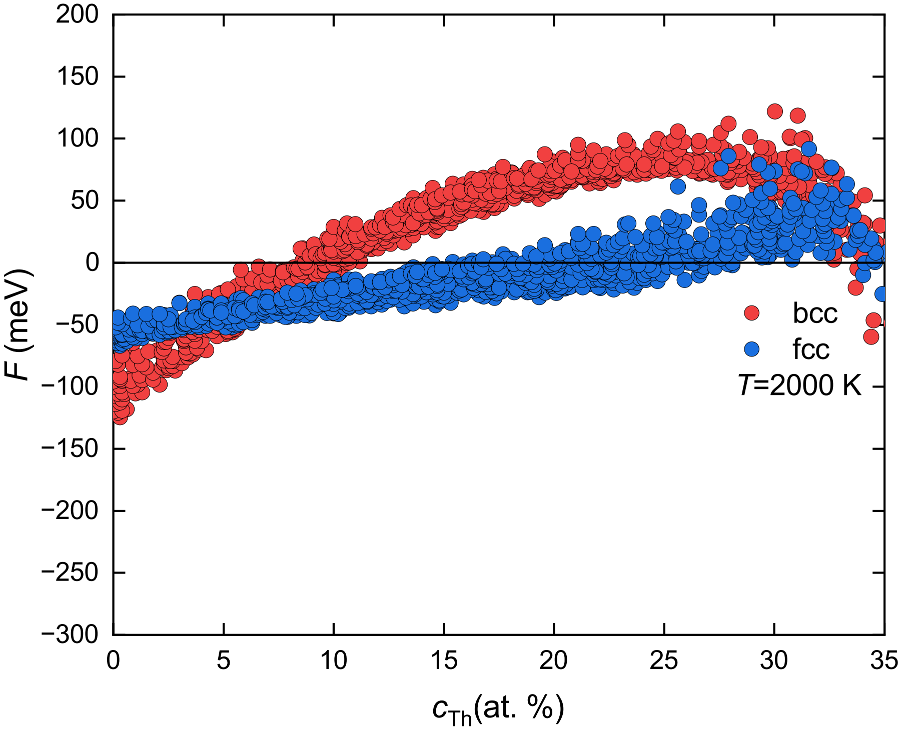

Figure S3 Free energy changes calculated for both bcc and fcc phases in as a function of Th concentration at the temperature of 2000 K.

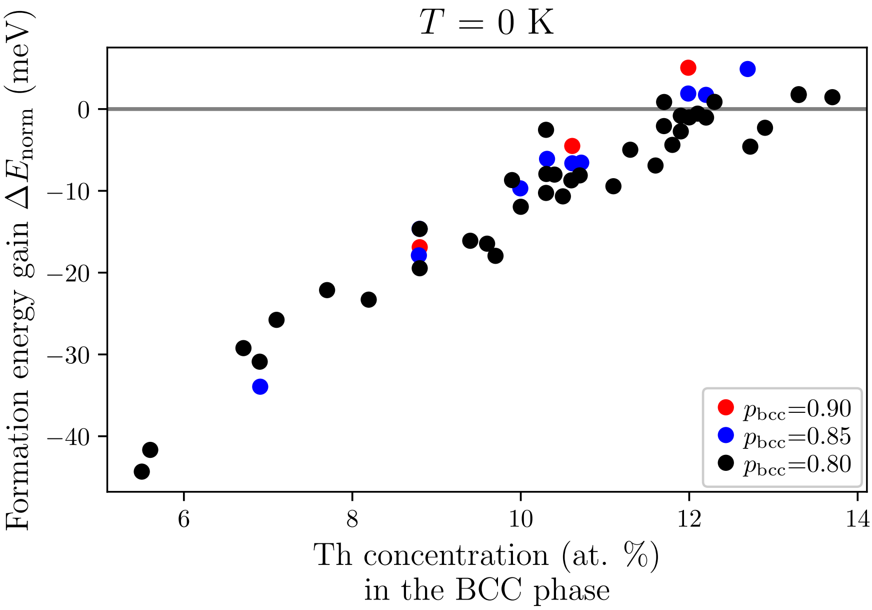

Figure S4 The energy gain ΔE_{nom} plotted as a function of Th concentration in the BCC phase calculated at the temperature of 0 K.

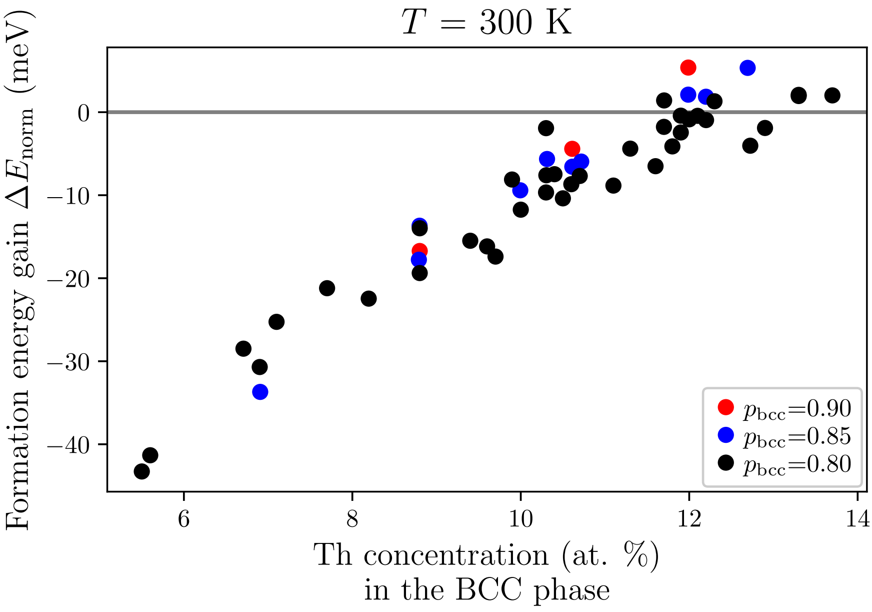

Figure S5 The energy gain ΔE_{nom} plotted as a function of Th concentration in the BCC phase calculated at the temperature of 300 K.

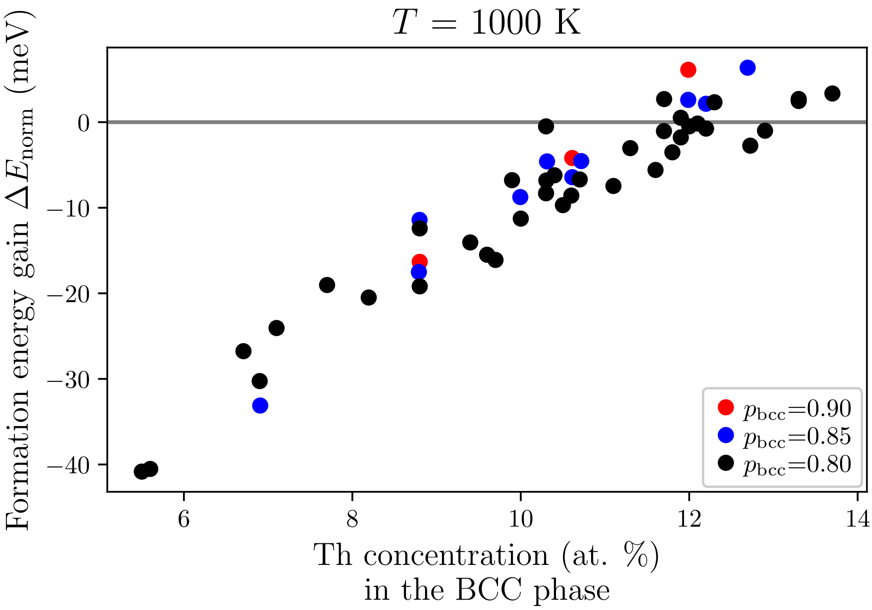

Figure S6 The energy gain ΔE_{nom} plotted as a function of Th concentration in the BCC phase calculated at the temperature of 1000 K.

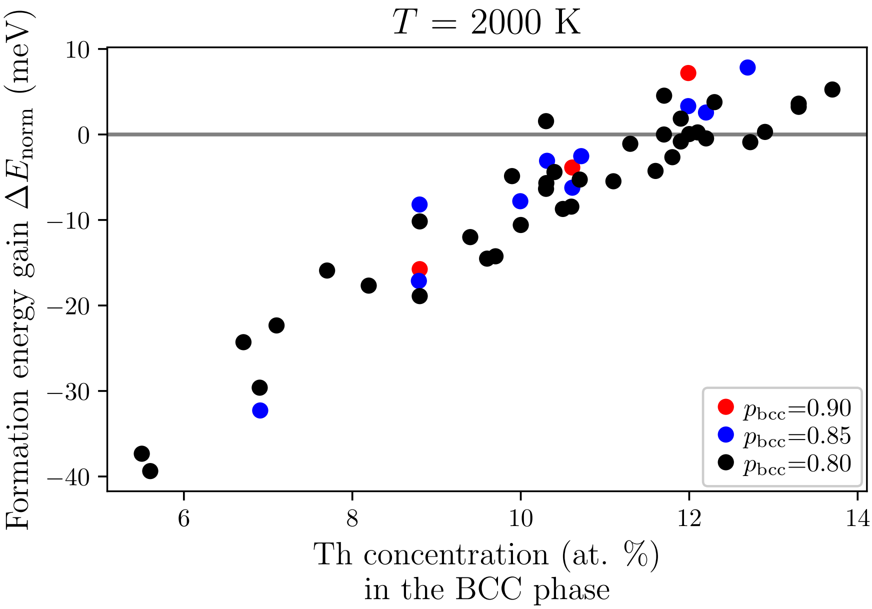

Figure S7 The energy gain ΔE_{nom} plotted as a function of Th concentration in the BCC phase calculated at the temperature of 2000 K.
